# Spinning magnetic field patterns that cause oncolysis by oxidative stress in glioma cells

**DOI:** 10.1038/s41598-023-46758-w

**Published:** 2023-11-07

**Authors:** Shashank Hambarde, Jeanne M. Manalo, David S. Baskin, Martyn A. Sharpe, Santosh A. Helekar

**Affiliations:** 1 Kenneth R. Peak Center for Brain and Pituitary Tumor Treatment and Research, Houston Methodist Hospital, Houston, TX USA; 2 Department of Neurosurgery, Houston Methodist Hospital, Houston, TX USA; 3 Houston Methodist Research Institute, Houston, TX USA; 4 Department of Neurosurgery, Weill Cornell Medical College, New York, NY USA

**Keywords:** Cancer, Oncology

## Abstract

Raising reactive oxygen species (ROS) levels in cancer cells to cause macromolecular damage and cell death is a promising anticancer treatment strategy. Observations that electromagnetic fields (EMF) elevate intracellular ROS and cause cancer cell death, have led us to develop a new portable wearable EMF device that generates spinning oscillating magnetic fields (sOMF) to selectively kill cancer cells while sparing normal cells in vitro and to shrink GBM tumors in vivo through a novel mechanism. Here, we characterized the precise configurations and timings of sOMF stimulation that produce cytotoxicity due to a critical rise in superoxide in two types of human glioma cells. We also found that the antioxidant Trolox reverses the cytotoxic effect of sOMF on glioma cells indicating that ROS play a causal role in producing the effect. Our findings clarify the link between the physics of magnetic stimulation and its mechanism of anticancer action, facilitating the development of a potential new safe noninvasive device-based treatment for GBM and other gliomas.

## Introduction

Reactive oxygen species (ROS) levels in cells play diverse roles in normal cellular processes such as developmental cell proliferation and differentiation, programmed cell death, cell motility, immune defense mechanisms, inflammation, and neuronal activity and plasticity^1–8^. They are also involved in cancer cell proliferation and tissue invasion, on the one hand, and cellular aging and neurodegeneration, as mediators of oxidative stress, on the other^9–12^. Cancer cells are known to possess high levels of ROS because of increased oxidative metabolism and dysfunctional mitochondria^9–11^. Previous studies have shown that abnormally high levels of ROS cause apoptosis. Therefore, it has been proposed that increasing ROS in cancer cells by drugs might have a role in the treatment of cancer^13,14^. Several existing anticancer drugs which have been shown to induce ROS might be acting in part through this mechanism^13,15^.

Besides drugs, stimulation by electromagnetic field (EMF) generating devices has been shown to raise ROS levels in cancer cells and thereby induce cell death of malignant tumor cells in vitro^16–20^. While some of these devices have shown safety and efficacy in mouse tumor xenograft models, to our knowledge no large patient trials have been conducted to date. In human cancer cells they produce variable results, showing both increases^21–25^ and decreases in ROS levels^26–28^, as well as a lack of change^26–30^ in them. This is likely due to the variability in the EMF-generating electromagnetic coils, experimental conditions, and cell types across studies^31^. Their underlying biophysical mechanism of action is not clear. Additionally, the precise range of physical parameters of EMF that produce a potentially therapeutic increase in ROS levels has not been fully characterized. Our recently developed noninvasive EMF device addresses these limitations because its stimulus parameters can be better and more precisely controlled and targeted. It generates a spinning oscillating magnetic field (sOMF or OMF) by rapidly rotating strong neodymium permanent magnets^32^. We have shown that this device, referred to as the “Oncomagnetic” device, substantially and consistently raises ROS in patient derived glioblastoma (GBM) cells to levels that are selectively cytotoxic to these cells, while sparing normal cells^33^. sOMF does not kill cultured normal human developing neurons, alveolar epithelial cells, and astrocytes^33^. We have also demonstrated recently that daily 2-h 3 times a day sOMF stimulation of normal wild-type mice for 4 months does not cause any adverse effects or abnormal histopathological changes in theirs tissues^34^. Furthermore, we have obtained evidence for the safety and efficacy of the device in mice implanted with orthotopic GBM xenografts^35^ and in an end-stage patient with recurrent GBM with no standard of care treatment options^32^. We have tested and found support for the hypothesis that the sOMF-induced increase in ROS is likely due to perturbation of the electron transfer process in the mitochondrial electron transport chain (ETC)^36^. Consistent with this hypothesis we have also found that sOMF inhibits the activity of Complex II succinate dehydrogenase^36^. We proposed this Magnetic Electron Perturbation (MEP) hypothesis because of the well-known effect of weak (< 1 mT) and intermediate range (1–10 mT) magnetic fields on mixing of spins of unpaired electrons of free radical intermediates during the spin-correlated electron pairing process termed as the radical pair mechanism (RPM)^37–41^. Potential influence of low strength magnetic fields on the ETC has been suggested earlier by others^41^.

Our Oncomagnetic device is programmable and allows precision control of all physical parameters of sOMF exposure (stimulation) both in vitro and in vivo, such as strength of the magnetic field (magnetic flux density), frequency of field oscillations, angles of rotation of the field axes, on and off intervals of intermittent stimulation, and duration and rate of stimulation. It therefore allows us to determine the exact parameters that consistently produce optimum increase in ROS. Furthermore, it also allows us to characterize the relationship between the field generated by the device and its interaction with the physical characteristics of RPM in the mitochondrial ETC protein complexes. For example, because these complexes have transmembrane orientations that are fixed in three dimensions over the time frame of sOMF pulse trains, we were able to investigate the possibility that changing the magnetic field axis angle in three dimensions is more effective at inducing ROS than static magnetic field distributed along one axis. Thus, in this study, we investigated the effects of varying each of the physical parameters that define the sOMF produced by the active components of the device called ‘oncoscillators’ in patient derived GBM and diffuse intrinsic pontine glioma (DIPG) cells.

## Results

### Effects of static magnetic field compared to sOMF

**Figure 1:**
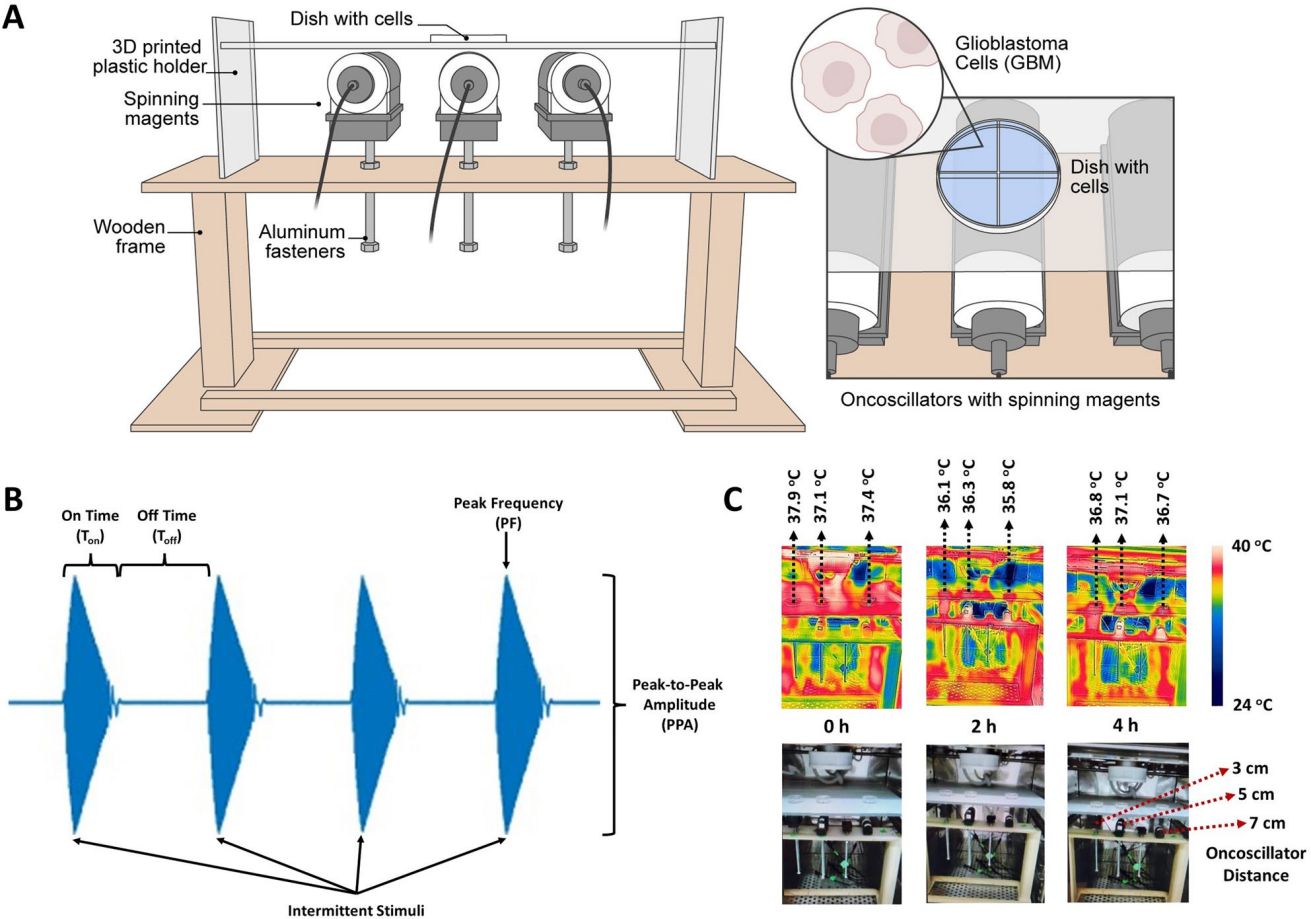
Cell culture stimulation setup, stimulation protocol and thermal imaging during sOMF stimulation to measure temperature changes. (**A**) Left—A schematic diagram of the cell culture sOMF stimulation setup used in the laboratory. Right—Closeup view of a cell culture dish placed above each oncoscillator. (**B**) A schematic diagram showing the stimulation protocol and indicating the stimulus parameters examined in our experiments. (**C**) Top Thermal images show false color-coded spatial temperature variations in the incubator at three time points during stimulation. Bottom Photographs of the apparatus and culture dishes corresponding to thermal images. To investigate whether the sOMF effects observed could be due to hyperthermia induced by stimulation we imaged the temperature of the culture dishes and the entire stimulation apparatus in the incubator during the 4-h stimulation session. To do this we used the FLIR One infrared thermal camera (Teledyne FLIR, Wilsonville, OR). We acquired images at the onset of stimulation (0 h) and at 2 h and 4 h time points during stimulation. We obtained 6 images at each time point and made spot measurements at the base of each culture dish placed at 3, 5 and 7 cm from the oncoscillator corresponding to PPA of ~ 5, ~ 1 and ~ 0.42 mT. We also measured the temperature at the base of a culture dish positioned at 1.4 cm from the oncoscillator corresponding to a PPA of ~ 58 mT and found no significant increase in temperature at this position.

**Figure 2:**
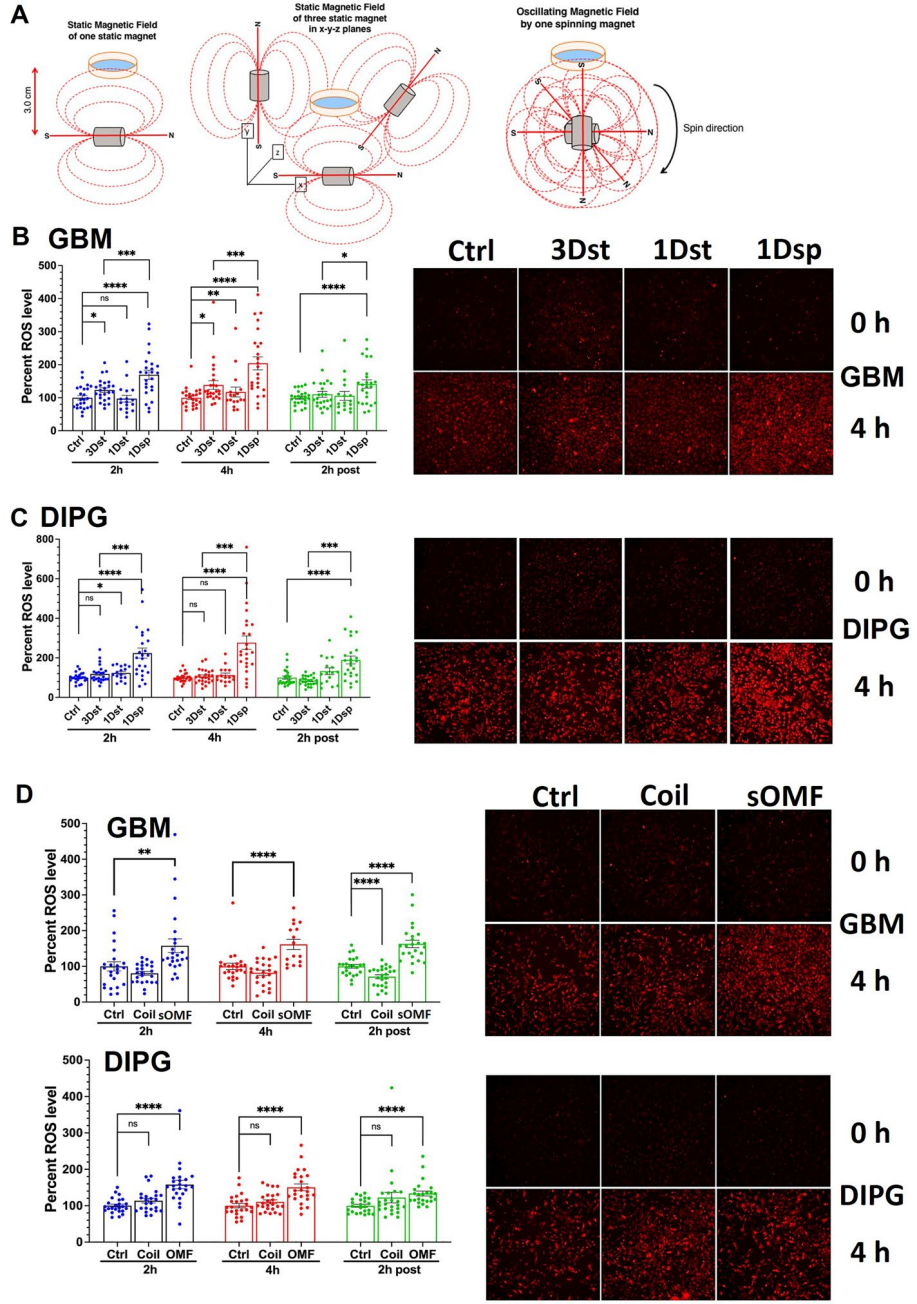
sOMF of spinning magnet generates higher cellular ROS than static magnetic field and both field oscillations and axis rotation are required for ROS induction. (**A**) Scheme for stimulation of cultured cells with static magnetic field of one stationary magnet, three stationary magnets along three orthogonal axes and oscillating magnetic field of one spinning magnet. (**B**) Fluorescence intensity quantitation of hydroethidine in GBM (GBM115) cells or (**C**) DIPG cells, from microscopic images (representative images shown on the right) at 2 h and 4 h during stimulation and at 2 h after the end of stimulation. Scatter with bars represent average normalized fluorescence intensity from three independent experiments with each data point shown as a dot (n = 24). Error bars depict standard errors of the mean (SEM). Ctrl—Unstimulated, 3DSt—stimulation with the magnetic field of three stationary magnets along the three orthogonal axes, 1Dst—one stationary magnet, and 1Dsp—one spinning magnet. ns *p* > 0.05, * *p* < 0.05, ** *p* < 0.01, *** *p* < 0.001, **** *p* < 0.0001. (**D**) Bar graphs with scatter showing cellular ROS levels in GBM115 (top) and DIPG (bottom) cells normalized to pre-stimulation control baseline during (2 h and 4 h) and 2 h post-stimulation corresponding to stimulation with the Helmholtz coil (Coil) and a single oncoscillator with magnet rotating in a 2-dimensional plane (sOMF), compared to unstimulated control (Ctrl). The stimulation was continuous for 4 h at ~ 137 Hz PF. Error bars show SEM. Representative microscope images of cells, before stimulation (0 h) and after 4 h stimulation, are shown in the right panel. ns *p* > 0.05, * *p* < 0.05, ** *p* < 0.01, *** *p* < 0.001, **** *p* < 0.0001.

As described above, we hypothesized that the interaction of weak and intermediate strength magnetic fields with the RPM mechanism in the mitochondrial ETC can perturb the electron transfer process (MEP hypothesis) to generate superoxide. The ETC membrane complex molecules are oriented in all directions and do not tumble, unlike molecules in solution. We predicted, therefore, that a spinning magnet should induce more ROS than a non-rotating magnet oriented along one fixed axis (1Dst). Furthermore, the static fields of three non-rotating magnets oriented along the three orthogonal axes in 3D space (3Dst) should also generate ROS comparable in amount to the spinning magnet (1Dsp). We tested these predictions by stimulating GBM and DIPG cells intermittently under all three conditions side by side using a specially designed experimental setup (Fig. 1A) and quantifying the fluorescence intensity of the superoxide indicator dye hydroethidine. The intermittent sOMF stimuli generated by an oncoscillator with a spinning magnet had a peak frequency (PF) of ~ 272 Hz, and on time (T_on_) and off time (T_off_) of 250 ms each (Fig. 1B). The oncoscillators did not produce an increase of temperature much beyond 37 °C at the location of stimulated cell culture dishes (Fig. 1C). The stimulation was carried out for 4 h. We observed that in both GBM and DIPG cells ROS generated by sOMF (1Dsp) was significantly higher than that generated by both 1Dst and 3Dst static magnetic fields at 2 h (during stimulation), 4 h (at the end of stimulation) and 6 h (2 h post-stimulation) (Fig. 2). While the increased effectiveness of a spinning magnetic field compared to a static field along one dimension confirms a prediction of the MEP hypothesis, the lack of a significant ROS-inducing effect of static fields oriented in all three dimensions suggests that the field oscillations themselves are essential for this effect.

### Effect of magnetic field oscillations along one fixed axis

Magnetic field oscillations induced by a rotating magnet are characterized by two distinct components – the sinusoidal waves of the magnetic field and the cyclically changing angles of the axis of the field. The above results indicate that field oscillations play a critical role in inducing ROS. To test whether oscillations are sufficient to produce the ROS effect we compared the effect of magnetic field oscillations produced by a Helmholtz coil, whose axis remains fixed in one orientation, with those produced by the rotating magnet of an oncoscillator.

The current passing through the coil alternated at ~ 137 Hz and was large enough to generate a peak-to-peak amplitude (PPA) of ~ 5 mT, values that were comparable to those produced by the spinning magnet of an oncoscillator used in this experiment. Both apparatuses delivered continuous stimulation for 4 h. We observed that Helmholtz coil did not produce any significant increase in ROS at 2 and 4 h during stimulation or 2 h post-stimulation in GBM and DIPG cells (Fig. 2D). In contrast, the oncoscillator significantly raised the ROS levels at all three time points in both cancer cell types. These observations indicate that an oscillating field alone is not sufficient to induce ROS and that the changing angle of the magnetic field axis is also required to achieve this effect.

### Comparison between intermittent and continuous sOMF

If the total amount of exposure to oscillations at the peak frequency is important for ROS induction, then we should expect a greater increase in ROS with continuous stimulation. Therefore, we examined whether there is a difference between continuous and intermittent sOMF stimulation with oncoscillators. Intermittent stimulation was delivered with T_on_ and T_off_ of 250 ms each. The PF for both types of stimulation was ~ 277 Hz. The bar plots in *Supplementary* Fig. S1A and B show that the level of ROS in cells exposed to continuous sOMF were not significantly higher than those exposed to intermittent sOMF in both GBM and DIPG cells. These data indicate that repeated pulse trains rising to and declining from the peak frequency with intervening pauses are sufficient to achieve near maximum level of increase in ROS.

### Magnet rotation along three axes

**Figure 3:**
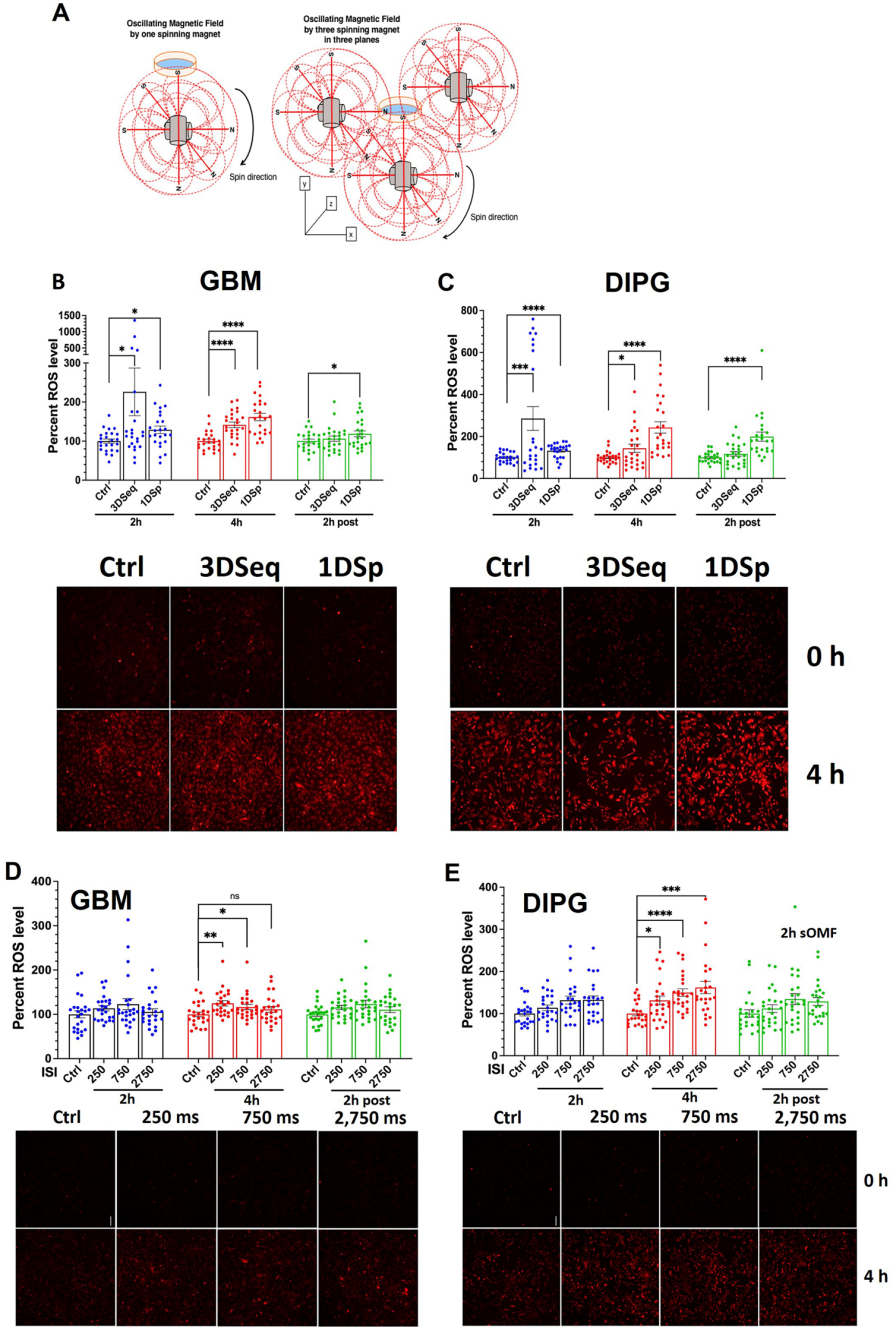
One spinning magnet or three spinning magnets generate similar cellular ROS levels and the effect of variation of the stimulus off period. (**A**) Scheme for stimulation of cultured cells with sOMF of one spinning magnet (1Dsp) with 250 ms T_on_ and 250 ms T_off_ or three spinning magnets (3Dseq) that are sequentially turned on and off (250 ms T_on_ and 250 ms T_off_). (**B**) Fluorescence intensity quantitation of hydroethidine in GBM (GBM115) cells or (**C**) DIPG cells using microscope images. Ctrl—Unstimulated. Representative microscopic images of cells before stimulation (0 h) and after 4 h of stimulation are shown below respective bar graphs. Data points, asterisks and error bars are as denoted in Fig. 1 (n = 24). (**D**) Fluorescence intensity quantitation of hydroethidine in GBM (GBM115) cells or (**E**) DIPG cells using microscopic images. Stimulations at different T_off_s are compared. Representative microscopic images of cells, before stimulation (0 h) and after 4 h stimulation, are shown below respective bar graphs. Data points, asterisks and error bars are as denoted in Fig. 1 (n = 24).

Because repeated changing of the angle of the magnetic field axis in all three dimensions may have a greater impact on ETC complexes oriented in all directions in space, we then investigated whether rotating magnets along all three orthogonal axes in three-dimensional space potentiates further the increase in ROS produced by magnet rotation along only one axis. We positioned three oncoscillators (3DSeq, Fig. 3A) at right angles to each other and activated them in repeating sequential or alternating cycles compared to intermittent stimulation with a single oncoscillator (1DSp). This experiment showed a greater increase in ROS at 2 h in both GBM and DIPG cells with 3DSeq compared to 1DSp stimulation; however, this difference was not statistically significant (Fig. 3B, C). The significant increases in ROS levels over control seen at 4 h and 2 h post-stimulation are also not significantly different between 3DSeq and 1DSp (Fig. 3B, C). This suggests that 1DSp stimulation might be sufficient to produce maximal ROS enhancement given that one activated oncoscillator sweeps through all angles in a two-dimensional plane.

### Variation in stimulus parameters

**Figure 4:**
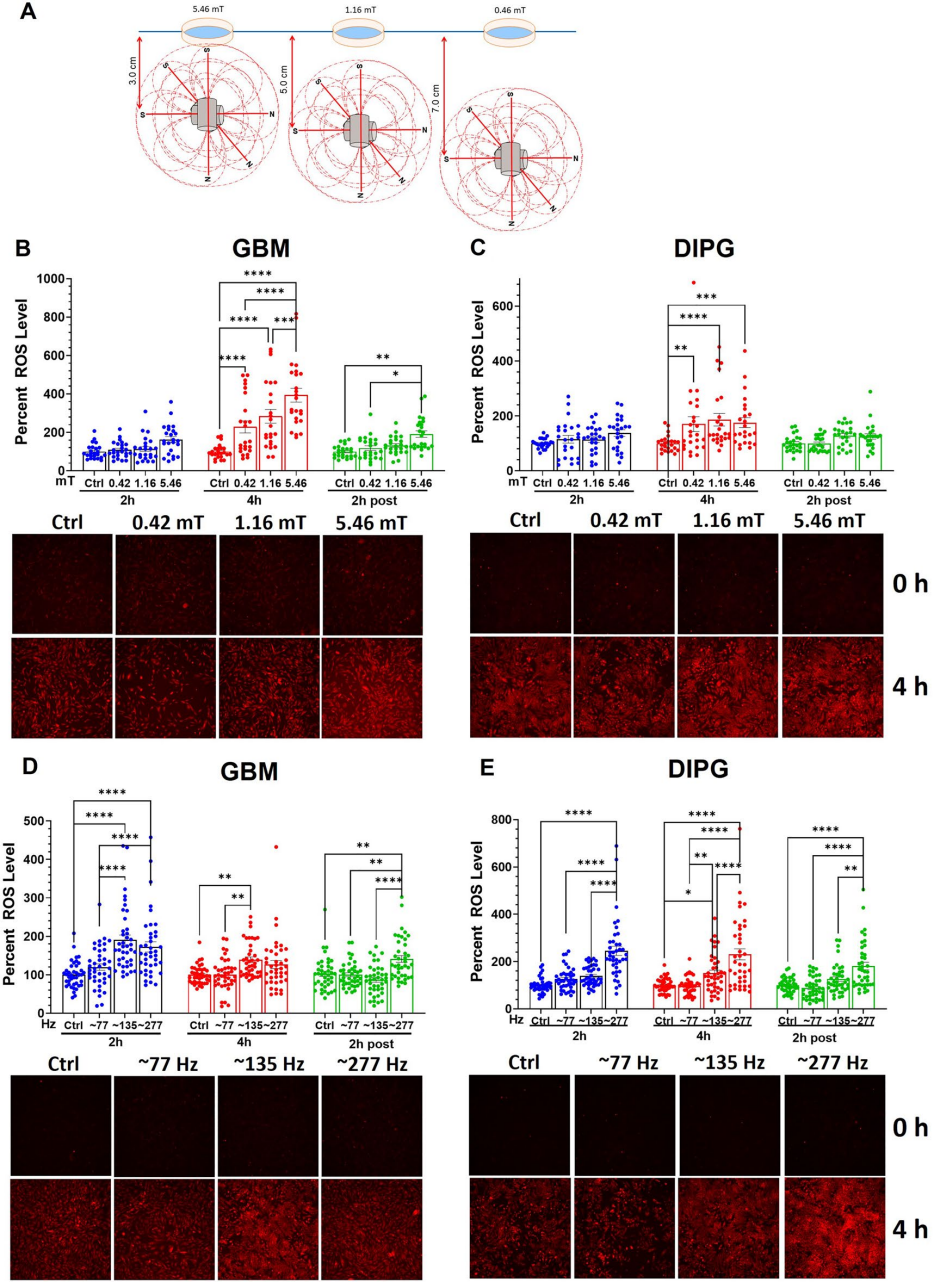
Effect of variation in magnetic field strength and peak frequency. The schematic in (**A**) depicts the positions of the cell culture dishes relative to the oncoscillators to obtain three different values of PPA or magnetic field strength. Bar graphs with scatter plots show the effect of variations in PPA (**B** and **C**), and PF (**D** and **E**). Data points, error bars and asterisks are as denoted in Fig. 1. (n = 24 and n = 40 for GBM and DIPG, respectively).

Stimulation with an oncoscillator has four physical parameters that can be varied along a continuous scale. They are T_on_, T_off_, strength of the magnetic field or PPA of the stimulus, and the stimulus PF. We kept T_on_ constant at 250 ms and determined the effects of varying the values of each of the other three parameters to at least three different levels. Comparing three different T_off_ values (250, 750 and 2750 ms) while keeping T_on_ at 250 ms (Fig. 3D, E) showed that in GBM cells 250 and 750 ms T_off_ produced slightly better effect than 2,750 ms on ROS generation (Fig. 3D). In contrast, in DIPG cells maximum ROS was generated by 2,750 ms T_off_ (Fig. 3E). We studied the effect of changing the PPA to ~ 0.42 mT, ~ 1.2 mT, ~ 5.5 mT and ~ 58.3 mT by positioning the cell culture dishes at distances of 7 cm, 5 cm, 3 cm, and 1.4 cm, respectively, from the oncoscillator (Fig. 4A–C and *Supplementary* Fig. S1C). All field strengths tested showed significant increases in ROS levels at the 4-h time point in GBM and DIPG cells (Fig. 4B, C). In terms of variation of PF between ~ 77, ~ 135 and ~ 277 Hz, the latter two frequencies were significantly more effective than ~ 77 Hz (Fig. 4D, E).

### Effect of repeated stimulation

To determine whether repeated sOMF stimulation produces a cumulative increase or any other effect on ROS levels we stimulated the cells for 2 h thrice at 2-h intervals. This stimulation pattern using optimum values of PPA, T_off_ and PF produced similar increases in ROS levels in both GBM and DIPG cells at each subsequent repetition compared to the first stimulation session (*Supplementary* Fig. S1D, E). This finding suggests neither potentiation nor desensitization of the ROS-inducing mechanism underlying the sOMF effect is produced by repeated epochs of stimulation with a spinning magnet.

### sOMF effects on cancer cell clonogenicity and caspase 3 activation, and their dependence on ROS induction

**Figure 5:**
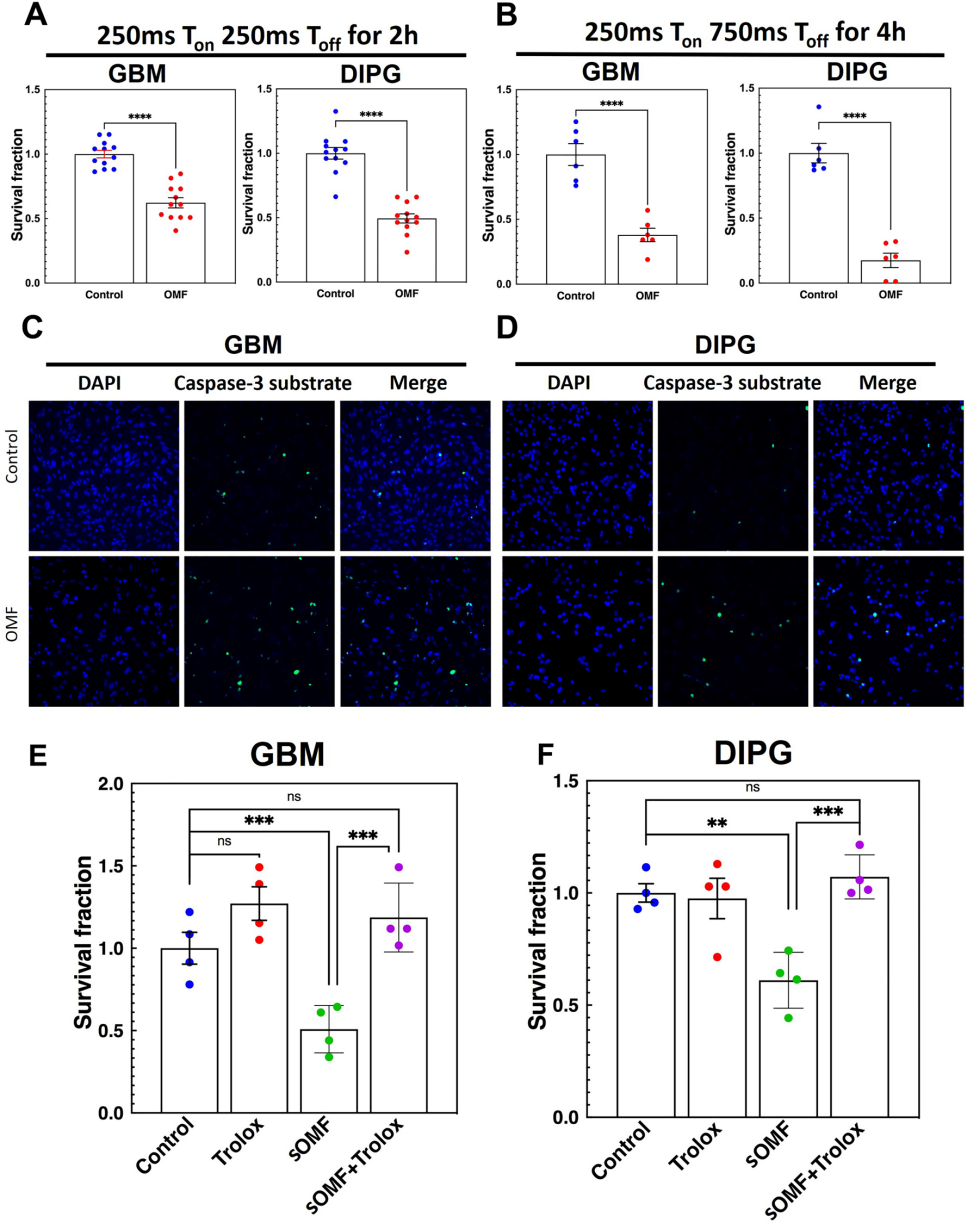
sOMF exposure causes ROS-dependent reduction in colony formation and cell death in GBM and DIPG cells. (**A** and **B**) Scatter with bar graphs show survival fraction in clonogenic cell survival assay for GBM (GBM115) and DIPG cells from independent experiments with each data point shown as a dot (n = 12). Error bars show SEM. Stimulation parameters are mentioned above bar graphs. (**C** and **D**) Representative images of caspase-3 activity increase 12 h after 4-h sOMF exposure in GBM and DIPG cells. (**E** and **F**) Scatter with bar graphs show survival fraction in clonogenic cell survival assay in the presence and absence of Trolox (20 µM) for GBM (GBM115) and DIPG cells (n = 4). Error bars show SEM. ** *p* < 0.01, ***p < 0.001, **** *p* < 0.0001.

Investigating various parameters of stimulation led us to identify the most effective range for the maximal induction of ROS in GBM and DIPG cells. Since higher ROS can cause oxidative damage to macromolecules and trigger cell death, we used the optimally effective parameters to stimulate GBM and DIPG cells and assess clonogenic cell survival and activation of caspase 3. The parameters in the optimum range used were intermittent mode of stimulation with PPA of ~ 5 mT, PF of ~ 137 Hz, T_on_ of 250 ms, T_off_ of 750 ms and stimulation duration of 2 h or 4 h. We performed a standard clonogenic cell survival assay with the optimized parameters to find out whether and how much reduction in the survival fraction is caused by 2-h and 4-h sOMF stimulation. The 2-h stimulation had a T_off_ of 250 ms and 4-h stimulation of 750 ms. This corresponded to 28,800 and 19,200 pulse trains for 2-h and 4-h stimulations, respectively. With 2-h stimulation we observed > 60% and > 40% reduction in DIPG and GBM cell survival fraction with sOMF, respectively (Fig. 5A, *Supplementary* Fig. S2A). For 4-h stimulation, the respective values were > 80% and > 60% (Fig. 5B, Supplementary Fig. S2B, see *Supplementary* Fig. S3A, B for histograms of size distributions of colonies of all culture plates). We also observed survival fraction reduction in two other GBM cell lines, LN-18 and LN-229 (data not shown). This result is likely due to an immediate cytotoxic effect produced by short periods of sOMF stimulation on single cancer cells and a possible delayed cytostatic effect on them, in addition, to halt cell proliferation. Additionally, we also observed a modest increase in caspase 3 activation in both DIPG and GBM cells 12 h after the 4-h stimulation with optimum parameters (Fig. 5C, D). Finally, we tested if sOMF-induced cell death is primarily via ROS generation by adding an antioxidant in the culture media in the survival assay. We exposed GBM and DIPG cells to sOMF in the presence of Trolox, a water-soluble form of vitamin E. The presence of Trolox completely rescued both GBM and DIPG cells from the cytotoxic and anti-proliferative effects of sOMF and enhanced the surviving fraction to the untreated control level (Fig. 5E, F, Supplementary Fig. S2C, D). This strongly suggests that sOMF-induced cell death and anti-proliferative effect depend primarily on cellular ROS generation.

## Discussion

In this study, we tested the MEP hypothesis stated above, and determined the effectiveness of a range of sOMF stimulation parameters in inducing the superoxide component of ROS in human GBM and DIPG cells. We then studied whether sOMF stimulation with an optimized set of parameters produces high anticancer potency in standardized assays. We find that concurrent exposure to oscillations of a magnetic field produced by rotations of its axis in a two-dimensional plane cause a maximal rise in the superoxide component of ROS in human GBM and DIPG cells. A set of values chosen for 2-h and 4-h sessions of stimulation from the optimum range of parameters are effective in killing GBM and DIPG cells and reducing the fraction of surviving cells in colonies in a clonogenic cell survival assay. Even though the total number of pulse train stimuli of the same duration (250 ms) applied over 4 h (19,200) is less than that applied over 2 h (28,800), 4-h stimulation produces a more pronounced suppression of the growth of GBM and DIPG cell colonies. This indicates that the total amount of energy delivered to cancer cells is clearly not the determinant of the potency of stimulation. Instead, it appears that the longer T_off_ between stimuli of 750 ms in the 4-h stimulation, as opposed to 250 ms in the 2-h stimulation might be the critical variable. Alternatively, repeated stimulation over a longer period might be responsible for the stronger effect. Mitochondria are the predominant site of production of ROS^1,42,43^, and the longer T_off_ might be important for the superoxide generated to trigger the loss of mitochondrial integrity, as shown by Sharpe et al.^36^, although extra-mitochondrial sources such as cryptochromes cannot be ruled out^1,2^. The fact that the oncolytic effect is mediated by an increase in ROS is strongly supported by the observation that sOMF-induced reduction of surviving cancer cell colonies is completely reversed by the antioxidant Trolox, which quenches superoxide and hydrogen peroxide^44^. The biological effects cannot be accounted for by stimulation-induced hyperthermia because exposure to sOMF generated by the oncoscillators does not cause a rise in temperature at the separation distances between their magnets and the stimulated cell cultures used in our experiments (Fig. 1C).

There is substantial literature on the effects of EMF on cells, in general, and on cancer cells, in particular. Excluding EMF methods that involve hyperthermia or thermal ablation and electroporation for diffusion of anticancer drugs, cytotoxic effects of electric and magnetic fields on cancer cells have been attributed to various intracellular and cell membrane-based mechanisms^16,45^. Most studies have documented changes in ROS levels; however, the direction and extent of these changes have tended to be highly variable^17,31^. A predominant reason for this variability seems to be the wide range of physical methods and parameters used in exposing cells to EMF. Preclinical and clinical studies involving EMF have primarily used either electromagnetic coils to induce magnetic fields or electrodes to generate electric fields. However, there are a few reports on treatment of cancer cells in culture and tumor xenografts in mice, with slowly rotating (up to 7 Hz) large permanent magnets, that show anticancer responses. These studies have not delved into the exact underlying mechanisms of action and have not investigated the involvement of ROS^46–50^. A non-portable equipment involving slow rotation of large neodymium magnets has also been used to treat non-small cell lung cancer patients in a pilot clinical trial^51^.

Devices or methods producing magnetic fields with electromagnetic coils for anticancer studies include Pulsed EMF (PEMF) generators, Bio-Electro-Magnetic-Energy-Regulation (BEMER) system, Thomas-EMF pattern-inducing coils, extremely low frequency EMF (ELF-EMF) delivering apparatuses and amplitude-modulated coil devices such as the Therabionic device^16,20,52–54^. They deliver magnetic fields with flux densities in the low µT or low mT range. While some of them have been shown to raise ROS levels in cancer cells, their physical basis and the exact underlying mechanism have not been explored. Furthermore, the studies have also concentrated on other mechanisms, such as calcium influx through L and T type calcium channels, not recognized to be directly linked specifically to cytotoxic mechanisms^53,55,56^. Two main technologies producing electric fields investigated for cancer treatment are tumor treating fields (TTF) or Optune^®^ alternating electric field-producing arrays and nanosecond Pulsed Electric Field (nsPEF) devices^57–59^. TTF is thought to disrupt microtubule spindle elements and mitotic chromosomal order during exit from metaphase^60^ by interfering with proteins possessing high dipole moments from surface electric charge separation, such as α/β-tubulin monomers and the mitotic septin complexes^61,62^. Effects on immune mechanisms have also been postulated for TTF^63,64^. nsPEF application is shown to produce nanopores in the membranes of cancer cells and intracellular organelles leading to cell death^65^; however, the mechanisms underlying these effects are unclear and their selectivity with respect to cancer cells has not been established^59,66^.

Of these EMF-based devices, only the Optune^®^ device has been approved by the U. S. FDA after successful safety and efficacy studies for the treatment of recurrent and newly diagnosed GBM^67,68^, and more recently for unresectable pleural mesothelioma^69^. As stated above, the proposed mechanism of action of this device differs entirely from that of our sOMF-producing device. Unlike in the case of sOMF stimulation, in which as shown in this study, blockade of its ROS-dependent mechanism of action with Trolox reverses the cytotoxic effect on GBM and DIPG cells, no reversal of the effect has been demonstrated for TTF stimulation by blocking its proposed mechanism of action. While TTF generated by Optune^®^ have been incidentally shown to increase intracellular levels of hydrogen peroxide (H_2_O_2_)^70^, whether this is mitochondrial in origin or not has not been investigated. Furthermore, TTF treatment that shows an increase in H_2_O_2_ is reported to be 24 h in duration. Similarly, in contrast to > 60% and > 80% decreases in cell survival fraction in GBM and DIPG cells, respectively, with 4 h of sOMF stimulation in the present study, TTF stimulation of 3-day duration produces a decrease of ~ 10 to ~ 50% in three different types of GBM cell lines^62,71^. TTF treatment also produces a paradoxical increase in clonogenic survival in the U251 GBM cell line^71^. These differences indicate that underlying mechanisms of action between TTF and sOMF stimulations are clearly distinct from each other.

sOMF induction of ROS indicates that the immediate targets of its action are redox mechanisms in cells. Magnetic fields at or above 1 mT are known to alter the kinetics and yields of certain chemical reactions involving free radical intermediates exchanging unpaired electrons^72,73^. These effects are now recognized in the context of RPM to arise from the conservation of electron spins in radical recombination reactions and long-lasting spin coherences of spin-correlated electrons in radical pairs (*Supplementary* Fig. S3A, B).

This study demonstrates that sinusoidal magnetic field oscillations combined with rapidly changing angles of the magnetic field axis in defined frequency and timing patterns at low mT flux densities can induce a rapid increase in the superoxide component of ROS in human GBM and DIPG cells in culture, trigger their death and prevent the growth of their colonies. The bridge between these biological effects and the physical influence of magnetic field appears to be quantum effects involving RPM underlying electron transfer reactions in the mitochondrial respiratory chain. This hypothesis needs to be rigorously tested in future experiments. However, the present experimental results provide a rational basis for employing sOMF produced by spinning permanent magnets for the noninvasive treatment of GBM, DIPG and other solid malignancies, as has been done by us recently in a case report describing the first-in-human compassionate use treatment of a patient with end-stage recurrent GBM^32^. Ongoing and planned experiments in our laboratory are directed to investigating the safety and efficacy of sOMF oncomagnetic therapy in syngeneic and orthotopic xenograft mouse models of glioma and the underlying mechanisms of device action. We are also continuing a study involving compassionate use oncomagnetic treatment in end-stage recurrent glioma patients. sOMF therapy may provide a new and effective treatment for highly malignant and lethal cancers.

## Methods

All methods used in this study were carried out in accordance with relevant guidelines and regulations**.**

### Cell lines and reagents

GBM115 is a Temozolomide-resistant cell line that was derived from GBM patient tissue resected by DSB. Its collection and research use were approved by Houston Methodist Research Institute institutional review board and an informed consent was obtained from the patient. It was authenticated by the University of Arizona Genetics Core (Tucson, Az). Its O-6-methylguanine-DNA methyltransferase (MGMT) methylation status is unmethylated and it has no epidermal growth factor receptor (EGFR) amplification. The DIPG cell line was purchased from Sigma-Aldrich and GBM cell lines LN-18 and LN-229 from ATCC. Cancer cell lines were cultured in DMEM supplemented 10% fetal bovine serum, 11 mM glucose and penicillin and streptomycin antibiotics in humidified incubator with 5% CO_2_ at 37 °C. All sOMF exposures as well as control sets were carried out in a humidified incubator with 5% CO_2_ at 37 °C. Dihydroethidium (hydroethidine, Fisher Scientific) was dissolved in DMSO at 10 mM concentration (stored at − 20 °C) and diluted to 5 μM in culture media immediately before use.

### Static and oscillating magnetic field exposure

Oncoscillators were positioned at different distances from the cell culture plates to change the PPA of the sOMF felt by the cells to ~ 0.4, ~ 1, ~ 5 mT and ~ 58 mT using an apparatus specially constructed in house for this purpose (Fig. 1A). The values of magnetic flux density at the various distances from the axis of the axially magnetized cylindrical neodymium (N52) magnets used in the oncoscillators were measured using a Homend handheld digital WT10A gauss meter. Three different values of PF of rotation of the magnet (sOMF oscillation frequency) were used— ~ 77 Hz, ~ 135 Hz and ~ 277 Hz. For intermittent stimulation, the T_on_ or sOMF pulse train duration was kept constant at 250 ms. The T_off_ times were set at 250, 750 or 2,750 ms. All frequency and timing values were programmed into the microprocessor controlling the oncoscillators. sOMF effects produced by each set of parameters were compared with the other two sets and with unstimulated controls. Comparisons were also made with exposure to static magnetic field of one non-rotated magnet and three orthogonally oriented non-rotated magnets, as well as with the sOMF produced by rotating the latter three magnets. Stimulation with sOMF produced by a non-rotating Helmholtz coil was also carried out by passing a sinusoidal current of a strength sufficient to produce a PPA of ~ 5 mT in between its two solenoids where the cell culture dish was placed. The sinusoidal current was generated by a function generator (Wavetek, San Diego, CA) and amplified to the desired current amplitude by a high current amplifier (Taidacent, Shenzhen Taida Century Technology Co., Ltd., China). Cells were stimulated once for a total duration of 2 h or 4 h in each experiment. In one experiment they were stimulated for 2 h thrice with two 2-h intervals between the three stimulation periods to test for effects of repeated stimulations. Intermittent stimulation for 4 h was also compared with continuous stimulation for the same duration at the same PF and PPA.

### ROS detection and caspase 3 activation assays

Cells grown in glass bottom four-chamber dishes (one cell line in two chambers) were incubated with 5 μM hydroethidine for 30 min in the humidified incubator with 5% CO_2_ at 37 °C in dark. From each chamber 3–4 fluorescence images were randomly taken at every time point for each treatment group using Carl Zeiss microscope. Images before starting the static magnetic field or sOMF exposure (0 h), and after 2 h and 4 h of sOMF exposure were captured. The last image was taken 2 h after ending the sOMF exposure (2-h post). For caspase-3 activity detection, cells were incubated with NucView 488 dye for 30 min 12 h after 4 h of sOMF exposure. Cells were then fixed with 4% paraformaldehyde, permeabilized with Triton-X 100, and incubated with 4′,6-diamidino-2-phenylindole (DAPI) for 10 min before being imaged. Exposure time and magnification were kept the same for all images in all experiments.

### Clonogenic cell survival assay

GBM115 or DIPG cells were seeded 200 cells per dish for clonogenic assay and kept in a humidified incubator with 5% CO_2_ at 37 °C. After 8–10 h these dishes were transferred to an incubator in which the sOMF device was run. Following sOMF exposure dishes were transferred to cell culture incubator and were allowed to grow colonies for 10 days in the case of DIPG cells and 14 days in the case of GBM115 cells. Trolox (6-hydroxy-2,5,7,8-tetramethylchroman-2-carboxylic acid, 20 mM) was added in the culture media 30 min before exposure to sOMF and the colonies were allowed to grow in its presence. Colonies were fixed and stained with crystal violet stain (0.05% crystal violet, 1% formaldehyde, 1% methanol) for 15–30 min. Washed and dried colonies were manually counted.

### Data acquisition and statistical analysis

We performed separate experiments to test each variable independently. Unmodified fluorescence micrographs, stained with hydroethidine at 10× resolution, were digitized on a desktop computer interfaced with the microscope and imported into the MATLAB programming environment (Mathworks, Natick, MA). MATLAB scripts written in house were used to automatically count RGB-encoded red cell pixels with fluorescence intensity above a uniform threshold setting. The intensity values obtained were normalized with respect to T0, i.e., pre-stimulation time point in each stimulation condition and the first time point in the no stimulation condition, or the average of all T0 values. The normalized value at each time point in the stimulation conditions was then renormalized with respect to the normalized value at the corresponding time point in the no stimulation condition. Data were pooled from three repetitions of each experiment and two-tailed Student’s pooled t test with false discovery rate test for multiple comparisons was used to assess statistical significance at *p* = 0.05 level. Bar graphs in the figures are presented with scatter to show variation in data between samples and experiments. Error bars represent standard errors of the mean (SEM). Survival fraction was calculated by dividing the number of colonies by number of cells seeded and normalized with respect to the average survival fraction of the control in each respective experiment. The survival experiment was repeated, and average of experiments was plotted using the scatter with bars method of the Prism (version 9) software. Each dot in the scatter represents normalized survival fraction of one dish. Two-tailed Student’s pooled t test was used and false discovery rate test for multiple comparisons was applied as above. Statistical significance was set at *p* = 0.05.

### Supplementary Information

Supplementary Information.

## Data Availability

All data generated or analyzed during this study are included in this published article and its supplementary information file.
