# Supplementary material for: Spinning magnetic field patterns that cause oncolysis by oxidative stress in glioma cells
Source: Sci Rep. 2023 Nov 7;13:19264. doi: 10.1038/s41598-023-46758-w (PMC10630398; doi:10.1038/s41598-023-46758-w)
Supplement: Supplementary file 1 — Supplementary Information. [file 41598_2023_46758_MOESM1_ESM.pdf]

Supplementary Information

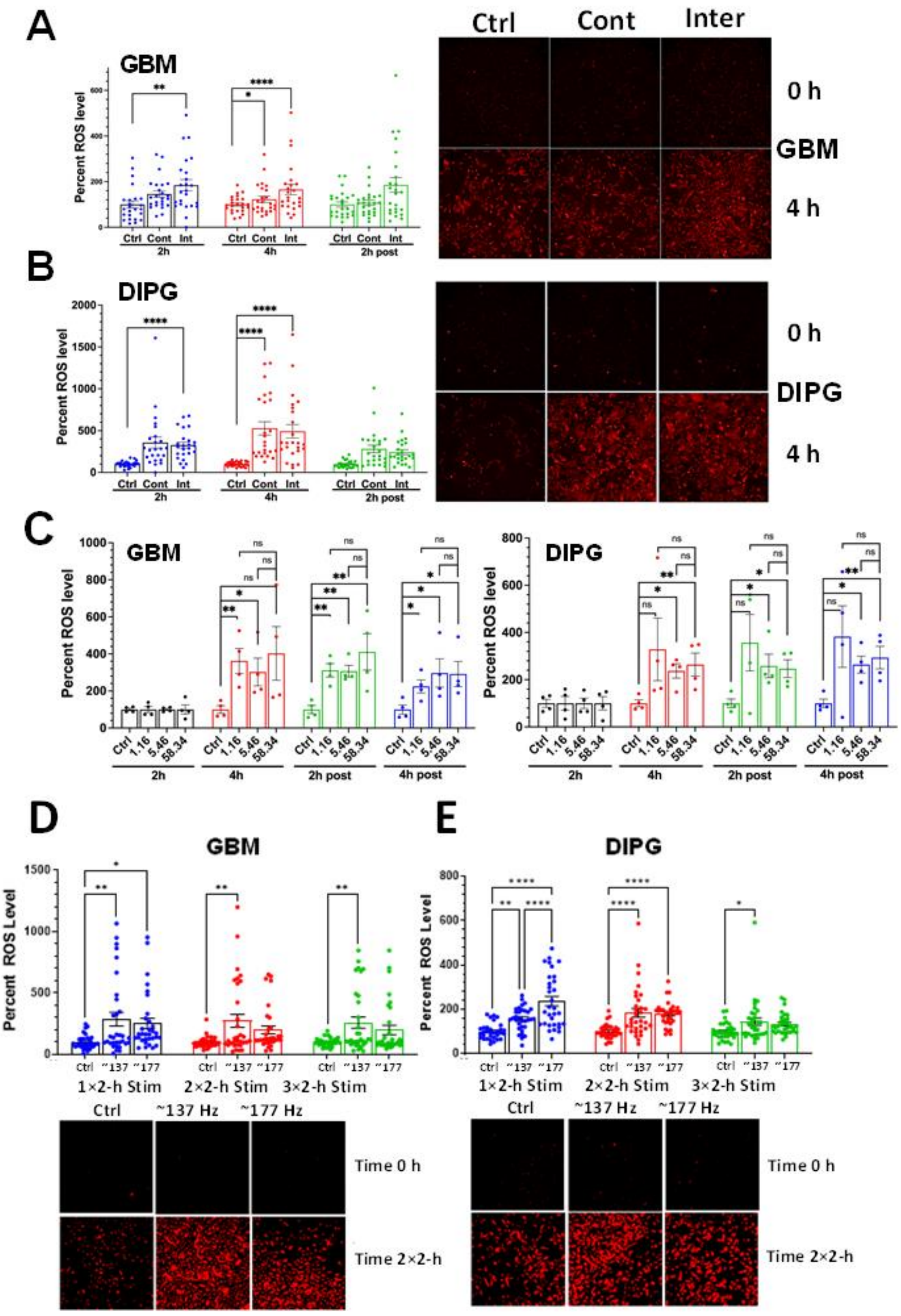

Fig. S1

Intermittent stimulation is as effective or better than continuous stimulation; increasing magnetic field strength from ~5 mT to ~58 mT shows no further ROS increase; and repeated sOMF stimulation with optimum parameters consistently induces similar increase in ROS.

Fluorescence intensity quantitation of hydroethidine in **(A)** GBM (GBM115) cells or **(B)** DIPG cells using microscope images. Conti – Continuously spinning magnet, Inter – magnet spinning intermittently for 250 ms (T_{on}) and stopping for 250 ms (T_{off}). Scatter with error bars (standard errors of the mean, SEM) represent average normalized fluorescence from three independent experiments with each data point shown as a dot (n=24). Representative microscopic images of cells before stimulation (0 h) and after 4 h of stimulation are shown in the right panel. ns $p > 0.05$, * $p < 0.05$, ** $p < 0.01$, *** $p < 0.001$, **** $p < 0.0001$. **(C)** Fluorescence intensity measurements of hydroethidine stained GBM (GBM115) and DIPG cells exposed to different magnetic field strengths. Scatter with bar graphs show mean of n=4 and error bars show SEM. The experiment was repeated, and representative data are shown. ns $p > 0.05$, *$p < 0.05$, **$p < 0.01$. **(D)** Fluorescence intensity quantitation of hydroethidine in GBM (GBM115) cells or **(E)** DIPG cells using microscopic images. Scatter with bars represent average normalized fluorescence intensity from three independent experiments with each data point shown as a dot (n=32). Error bars show SEM. Stimulation parameters are as indicated in each panel.

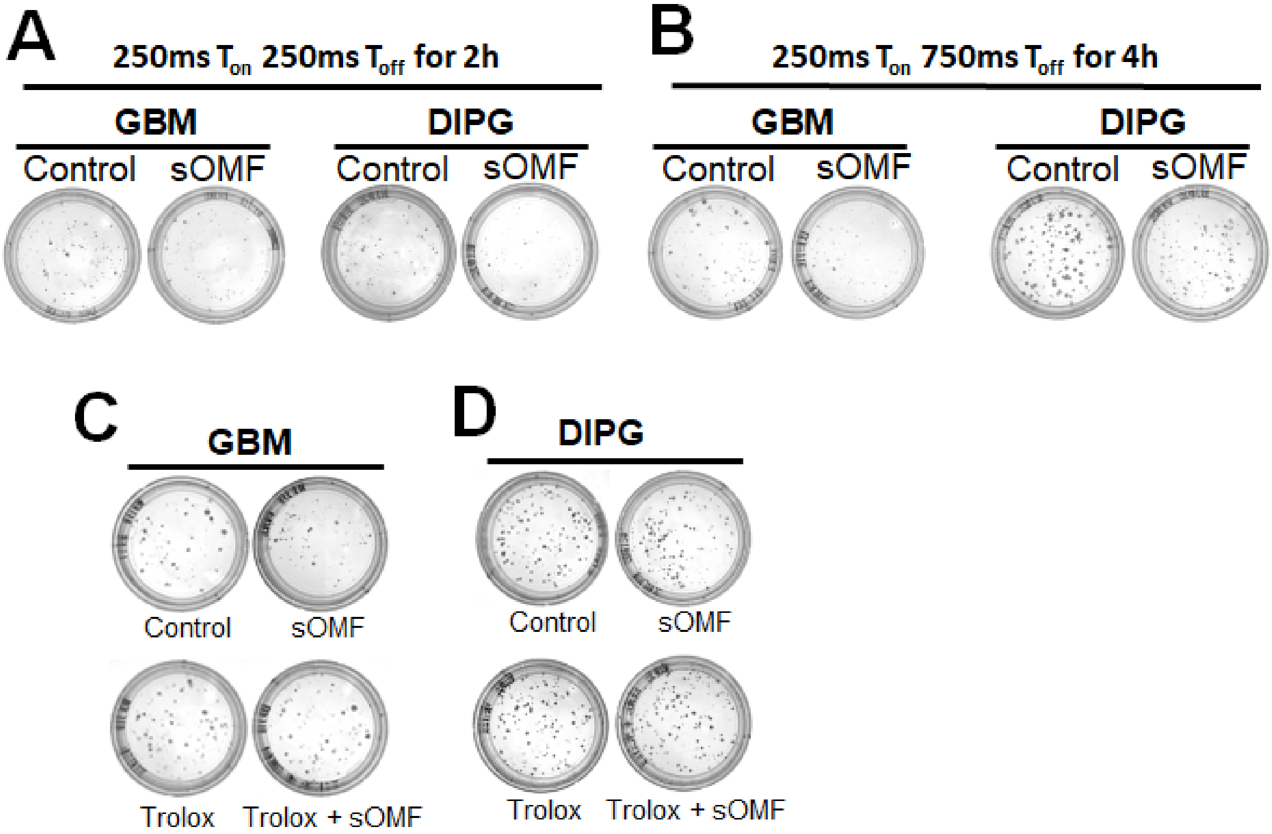

Fig. S2

Representative images of culture dishes from the clonogenic survival assays

(A) Representative images from the 2-h stimulation experiment **(B)** Representative images from the 4-h stimulation experiment. **(C)** Representative images from the Trolox rescue experiment in GBM cells. **(D)** Representative images from the Trolox rescue experiment in DIPG cells.

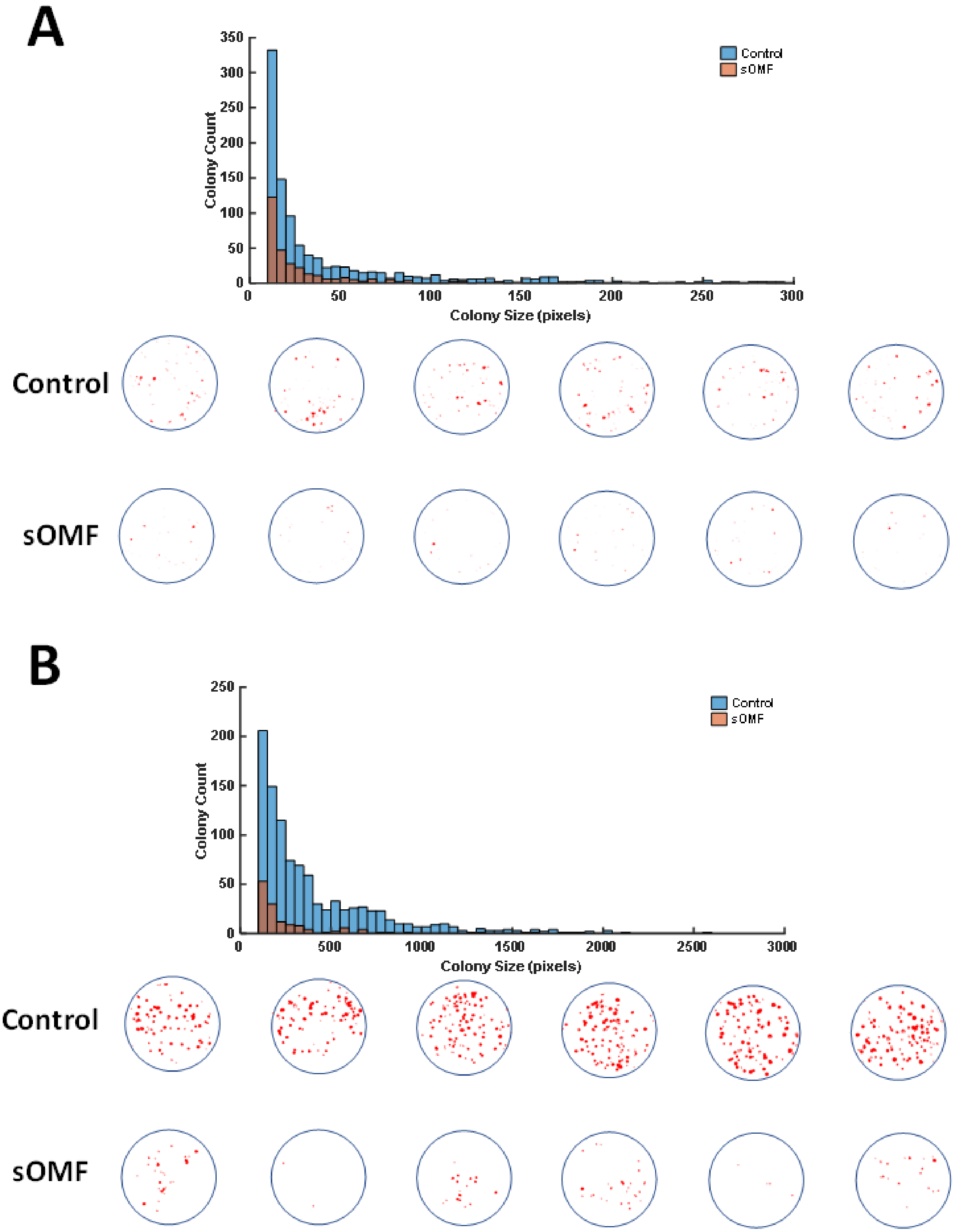

A
Colony Count
Colony Size (pixels)
Control
sOMF
350
300
250
200
150
100
50
0
0
50
100
150
200
250
300
Control
sOMF
B
Colony Count
Colony Size (pixels)
Control
sOMF
250
200
150
100
50
0
0
500
1000
1500
2000
2500
3000
Control
sOMF

Fig. S3

Size distribution of GBM and DIPG cell colonies

(A) Top Histogram of GBM115 cell colony sizes measured and plotted using an in-house MATLAB program. **Bottom** Representations of colonies on all unstimulated (Control) and sOMF-stimulated (sOMF, 4-h stimulation) culture dishes displayed by the software program. There are reductions of colony counts at all colony sizes on dishes subjected to sOMF. **(B) Top** Histogram of DIPG cell colony sizes measured and plotted using an in-house MATLAB program. **Bottom** Representations of colonies on all unstimulated (Control) and sOMF-stimulated (sOMF, 4-h stimulation) culture dishes displayed by the software program. There are reductions of colony counts at all colony sizes on dishes subjected to sOMF. The MATLAB software program written in-house counts clusters of pixels corresponding to each colony in digitized images of culture plates and plots a histogram of colony count as a function of the colony size.

Proposed sOMF Effect on the Radical Pair Mechanism

RPM affected by magnetic flux density in the 1 – 10 mT range is characterized[1,2] by: (1) Reactants that produce a pair of radicals with electron spins in a singlet configuration, i.e., with antiparallel spins; (2) Spin-selective reaction of singlet pairs to produce singlet product or a non-selective back reaction; (3) Competition between these reactions and interconversion between singlet and triplet (parallel spins) pairs affected by an externally applied magnetic field (Fig. S4A); (4) The triplet pairs also reacting spin-selectively or back reacting non-selectively to give rise to triplet products; (5) In many instances, the non-selective product regenerating the initial reactants to restart the cycle; and (6) Singlet and triplet states affected by electron-nuclear hyperfine interactions.

In accordance with the MEP hypothesis, we propose that electron transfer processes in the ETC complexes might involve RPM, and therefore, might be perturbed optimally by sOMF in the observed ~1 – ~5 mT range (Fig. S4B). Our observation that there is no significant increase in the ROS induction effect at an order of magnitude increase in magnetic flux density (from ~5 mT to ~58 mT) is consistent with observations in the field of spin chemistry that the decrease in product yield in RPM reactions, or in the present case, the biological effect of increase in superoxide (O_2^-) plateaus in the flux density range of ~1 mT – >1 T (Fig. S4B)[3,4]. The involvement of magnetic field oscillations and the exact orientation of the axis of the field as critical determining factors in influencing RPM are also well documented[5], although the rationale for effectiveness of the super low frequency range in our experiments is not clear. The dependence of both these parameters together for the maximal induction of ROS in our experiments therefore supports the hypothesis that RPM is the target of sOMF. That rotation of a permanent magnet can achieve both makes this mode of magnetic stimulation particularly convenient. As an experimental confirmation of perturbation by sOMF of electron flow in the ETC, we have shown separately with oxygen electrode measurements that sOMF halts or retards the consumption of oxygen by isolated rat liver mitochondria and GBM and DIPG cells[6]. We have also observed that sOMF triggers the loss of mitochondrial integrity, which is blocked by bongkrekic acid and cyclosporin A[6]. It is known that superoxide opens membrane permeability transition pores in the inner mitochondrial membrane[7].

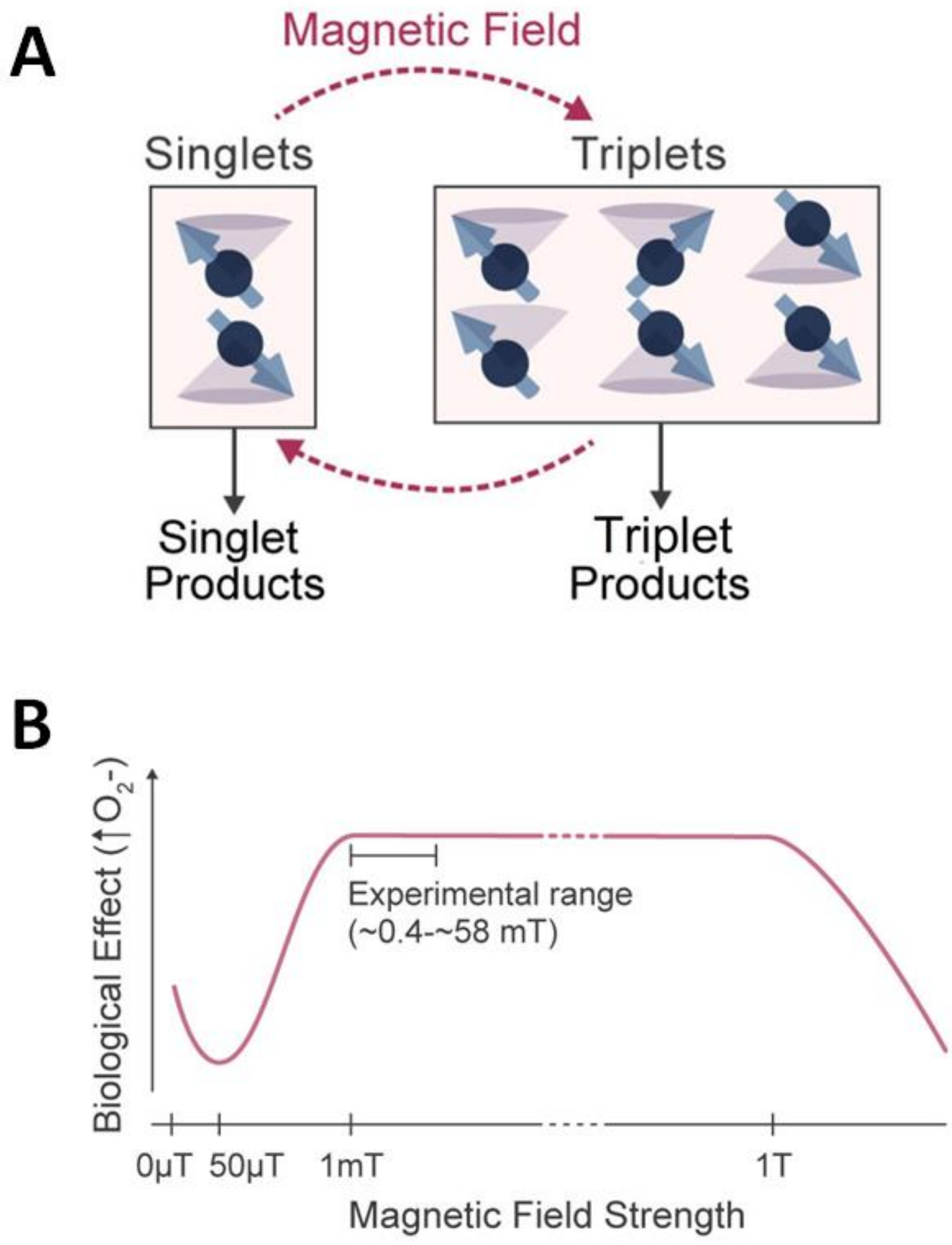
A
Magnetic Field
Singlets
Triplets
Singlet Products
Triplet Products
B
Biological Effect (↑O_2^-)
Experimental range
(~0.4-~58 mT)
0μT
50μT
1mT
1T
Magnetic Field Strength

Fig. S4

Representation of the influence of magnetic field on electron spins and representation of the hypothetical effect of magnetic field as a function of its strength

(A) Diagram showing magnetic field affecting the interconversion of electron spins from the singlet state to the triplet state. Vector representations of the spins are shown. **(B)** Diagram adapted in modified form from Woodward, 2002[3] showing that the effective range of the field strengths (~1 mT – ~58 mT) used in our experiments falls in the plateau phase of the biological effect. The arrow points in the increasing direction of the biological effect (i.e., increasing superoxide).

References

1 Rodgers, C. T. Magnetic field effects in chemical systems. *Pure Appl Chem* **81**, 19 - 43, doi:10.1351/PAC-CON-08-10-18 (2009).

2 Timmel, C. R., Till, U., Brocklehurst, B., McLauchlan, K. A. & Hore, P. J. Effects of weak magnetic fields on free radical recombination reactions. *Molecular Physics* **95**, 71-89, doi:10.1080/00268979809483134 (1998).

3 Woodward, J. R. Radical pairs in solution. *Prog. React. Kinet. Mech.* **27**, 165–207 (2002).

4 Woodward, J. R., Foster, T. J., Jones, A. R., Salaoru, A. T. & Scrutton, N. S. Time-resolved studies of radical pairs. *Biochem Soc Trans* **37**, 358-362, doi:10.1042/BST0370358 (2009).

5 Solov'yov, I. A. & Schulten, K. Magnetoreception through cryptochrome may involve superoxide. *Biophys J* **96**, 4804-4813, doi:10.1016/j.bpj.2009.03.048 (2009).

6 Sharpe, M. A., Baskin, D. S., Pichumani, K., Ijare, O. B. & Helekar, S. A. Rotating Magnetic Fields Inhibit Mitochondrial Respiration, Promote Oxidative Stress and Produce Loss of Mitochondrial Integrity in Cancer Cells. *Frontiers in Oncology* **11**, doi:10.3389/fonc.2021.768758 (2021).

7 Hirsch, T., Marzo, I. & Kroemer, G. Role of the mitochondrial permeability transition pore in apoptosis. *Biosci Rep* **17**, 67-76, doi:10.1023/a:1027339418683 (1997).
